# Crystal structure of tetra­aqua­[2-(pyridin-2-yl)-1*H*-imidazole-κ^2^ *N* ^2^,*N* ^3^]iron(II) sulfate

**DOI:** 10.1107/S2056989015004417

**Published:** 2015-03-11

**Authors:** Zouaoui Setifi, Fatima Setifi, Bojana M. Francuski, Sladjana B. Novaković, Hocine Merazig

**Affiliations:** a Laboratoire de Chimie, Ingénierie Moléculaire et Nanostructures (LCIMN), Université Ferhat Abbas Sétif 1, Sétif 19000, Algeria; b Unité de Recherche de Chimie de l’Environnement et Moléculaire Structurale (CHEMS), Université Constantine 1, Constantine 25000, Algeria; c Vinča Institute of Nuclear Sciences, Laboratory of Theoretical Physics and Condensed Matter Physics, PO Box 522, University of Belgrade, 11001 Belgrade, Serbia

**Keywords:** crystal structure, 2-(pyridin-2-yl)-1*H*-imidazole, iron(II) complex, hydrogen bonding, C—H⋯π inter­actions, π–π inter­actions

## Abstract

In the title compound, [Fe(C_8_H_7_N_3_)(H_2_O)_4_]SO_4_, the central Fe^II^ ion is octa­hedrally coordinated by two N atoms from the bidentate 2-(pyridin-2-yl)-1*H*-imidazole ligand and by four O atoms of the aqua ligands. The largest deviation from the ideal octa­hedral geometry is reflected by the small N—Fe—N bite angle of 76.0 (1)°. The Fe—N coordination bonds have markedly different lengths [2.1361 (17) and 2.243 (2) Å], with the shorter one to the pyrimidine N atom. The four Fe—O coordination bond lengths vary from 2.1191 (18) to 2.1340 (17) Å. In the crystal, the cations and anions are arranged by means of medium-strength O—H⋯O hydrogen bonds into layers parallel to the *ab* plane. Neighbouring layers further inter­connect by N—H⋯O hydrogen bonds involving the imidazole fragment as donor group to one sulfate O atom as an acceptor. The resulting three-dimensional network is consolidated by C—H⋯O, C—H⋯π and π–π inter­actions.

## Abstract 2

The Fe—O and Fe—N bond lengths in the title compound, [Fe(pyim)(H_2_O)_4_]SO_4_, where pyim is 2-(pyridin-2-yl)-1*H*-imidazole, are markedly different than in the related structure of [Fe(dmbpy)(H_2_O)_4_]SO_4_ where dmbpy is 5,5′-dimethyl-2,2′-bi­pyridine.

## Chemical context   

Polynitrile anions have recently received considerable attention in the fields of coordination chemistry and mol­ecular materials (Benmansour *et al.*, 2010 ▸). These organic anions are of inter­est due to their ability to act towards metal atoms with various coordination modes and for their high degree of electronic delocalization (Miyazaki *et al.*, 2003 ▸; Atmani *et al.*, 2008 ▸; Benmansour *et al.*, 2008 ▸, 2012 ▸; Setifi *et al.*, 2002 ▸, 2013 ▸, 2014 ▸; Addala *et al.*, 2015 ▸).
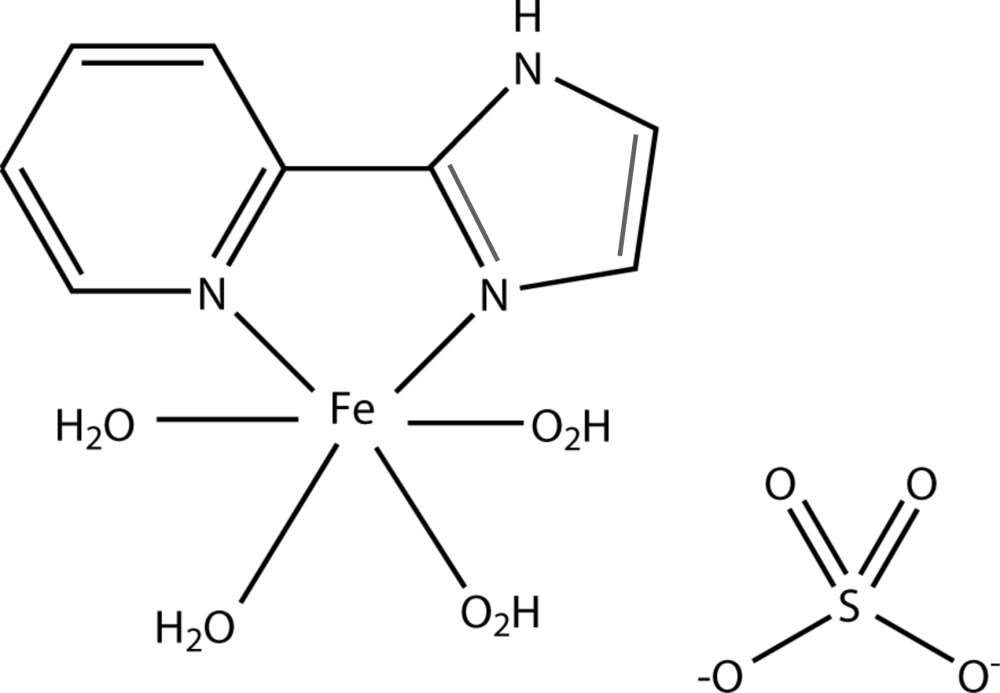


We are inter­ested in using these anionic ligands in combin­ation with other neutral bridging co-ligands to explore their structural features and properties relevant to the field of mol­ecular materials exhibiting the spin crossover (SCO) phenomenon (Dupouy *et al.*, 2008 ▸, 2009 ▸). In an attempt to prepare such an iron(II) complex using hydro­thermal synthesis, we obtained instead the title compound [Fe(pyim)(H_2_O)_4_]SO_4_, (I), where pyim is 2-(pyridin-2-yl)-1*H*-imidazole.

## Structural commentary   

**Figure 1:**
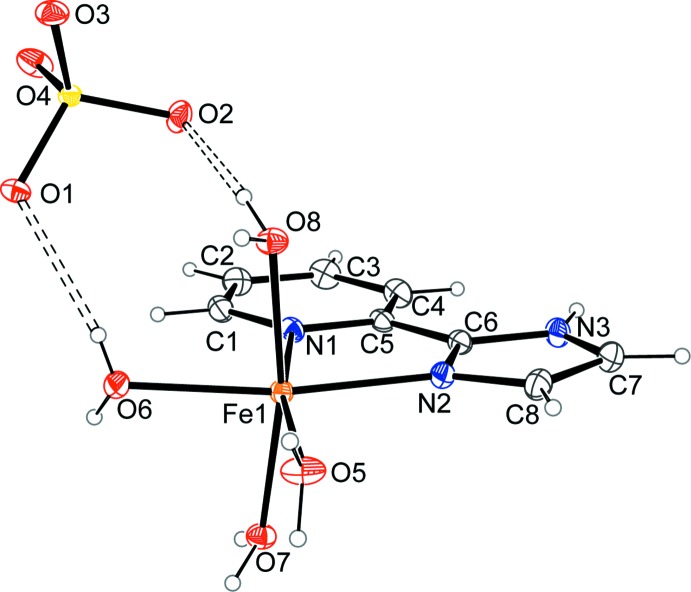
The mol­ecular structure of (I), with atom labels and displacement ellipsoids at the 50% probability level. Hydrogen bonds are shown as double dashed lines.

Fig. 1 ▸ shows the asymmetric unit of (I). The main building units in the crystal structure of (I) are octa­hedral [Fe(pyim)(H_2_O)_4_]^2+^ complex cations and [SO_4_]^2−^ anions. The distorted octa­hedral environment of the central Fe^II^ ion is defined by two N donor atoms of the pyim ligand and by the O atoms of two water mol­ecules in the equatorial plane, while the two remaining water mol­ecules coordinate at the axial sites. The bite angle N1—Fe—N2 of 76.04 (7)° shows the most significant deviation from the ideal octa­hedral geometry, with the other coordination angles deviating by 0.21 (7) to 11.91 (7)°.

The Fe—N coordination bonds with the chelate ligand have markedly different lengths, Fe—N1 = 2.243 (2) and Fe—N2 = 2.1361 (17) Å, which are also dissimilar to those in the previously reported [Fe(dmbpy)(H_2_O)_4_]SO_4_ complex where dmbpy is 5,5′-dimethyl-2,2′-bi­pyridine (Belamri *et al.*, 2014 ▸.) comprising a nearly symmetrical dipyridyl ligand [Fe—N = 2.176 (3) Å on average]. The torsion angles within the approximately planar five-membered chelate ring of (I) vary from 0.6 (3) to −5.2 (2)° and reflect a more pronounced deviation from planarity in comparison with the dmbpy Fe^II^ complex that exhibits a maximal torsion angle of 2.0 (3)°. The dihedral angle of 5.5 (1) ° between the aromatic rings of the pyim ligand is within the range of the values reported for the eight independent mol­ecules in the crystal structure of the non-coordinating ligand [1(1) to 17 (1)°; Tinant *et al.*, 2010 ▸]. In the present complex, all four Fe—O bond lengths, ranging from 2.1191 (18) to 2.1340 (17) Å, are longer than the corres­ponding ones in the [Fe(dmbpy)(H_2_O)_4_]SO_4_ complex, which range from 2.079 (2) to 2.110 (2) Å.

## Supra­molecular features   

**Figure 2:**
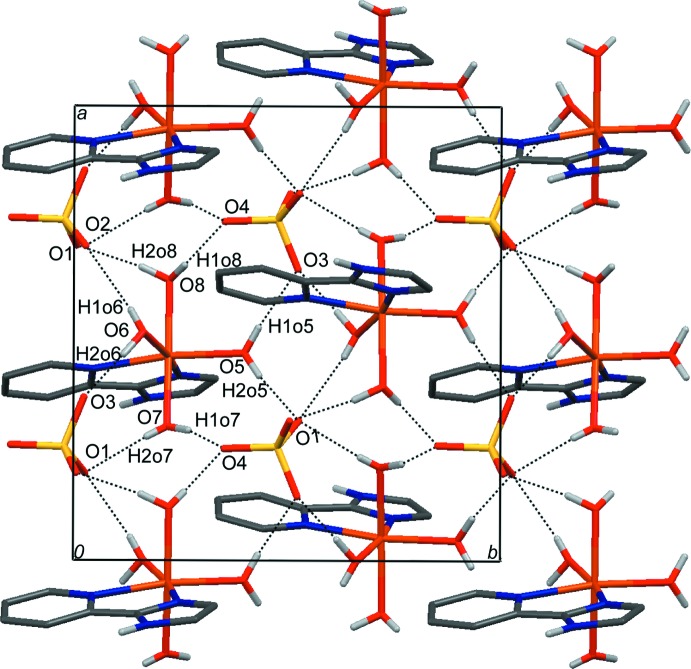
O—H⋯O inter­actions (dashed lines) connect cationic and anionic units into layers parallel to the *ab* plane (view of a single layer down the *c* axis). H atoms not involved in hydrogen bonding have been omitted for the sake of clarity.

**Figure 3:**
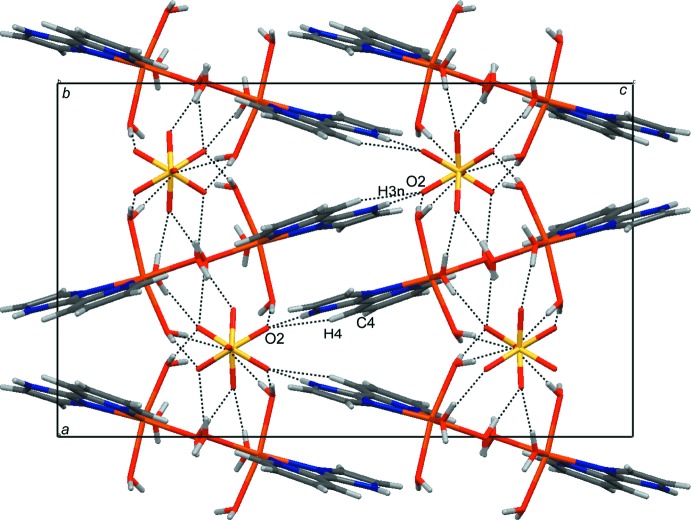
The three-dimensional packing of (I) viewed down the *b* axis.

The crystal packing of (I) is stabilized by a complex hydrogen-bonding network involving the coordinating water mol­ecules and the imidazole fragment as donors to the O acceptors atoms of the sulfate anion. Each cationic [Fe(pyim)(H_2_O)_4_]^2+^ unit is surrounded by five [SO_4_]^2−^ anions. Similarly to the crystal structure of [Fe(dmbpy)(H_2_O)_4_]SO_4_, pairs of axially and equatorially coordinating water mol­ecules bind to pairs of O acceptor atoms from the same [SO_4_]^2−^ group, forming eight medium-strength inter­actions (Table 1 ▸). These hydrogen bonds arrange the complex mol­ecules into layers parallel to the *ab* plane (Fig. 2 ▸). Additional N—H⋯O and C—H⋯O hydrogen bonds involving the donors from the aromatic ligand inter­connect adjacent layers into a three-dimensional arrangement (Fig. 3 ▸). The vicinity of aromatic rings in the inter-layer region gives rise to C—H⋯π [H3⋯*Cg*1^i^ = 3.033 Å; C3—H3⋯C*g*1^i^ = 117°; symmetry code: (i) = −*x* + 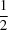, *y* + 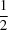, *z*; *Cg*1 is the centroid of the imidazole ring] and weak π–π inter­actions [*Cg*1⋯*Cg*2^ii^ = 3.821 Å, the shortest inter­atomic distance N3⋯C2^ii^ = 3.325 (1) Å; symmetry code: (ii) = −*x* + 1, −*y* + 1, −*z* + 1; *Cg*1 and *Cg*2 are the centroids of the imidazole and pyridine rings, respectively]. C—H⋯O inter­actions are also observed (Table 1 ▸).

## Synthesis and crystallization   

The title compound was obtained under hydro­thermal conditions from a mixture of iron(II) sulfate hepta­hydrate (28 mg, 0.1 mmol), 2-(pyridin-2-yl)-1*H*-imidazole (15 mg, 0.1 mmol) and potassium tri­cyano­methanide KC(CN)_3_ (26 mg, 0.2 mmol) in water-ethanol (4:1 *v/v*, 20 ml). The mixture was transferred to a Teflon-lined autoclave and heated at 423 K for 48 h. The autoclave was then allowed to cool to ambient temperature. Block-like yellow crystals of (I) were collected by filtration, washed with water and dried in air (yield 58%).

## Refinement details   

Crystal data, data collection and structure refinement details are summarized in Table 2 ▸. H atoms bonded to C atoms were placed at geometrically calculated positions and refined using a riding model. C—H distances were fixed to 0.93 Å for aromatic C atoms, with *U*_iso_(H) = 1.2*U*_eq_(C). The H atoms attached to O and N atoms were located in a difference Fourier map and were refined isotropically.

## Supplementary Material

Crystal structure: contains datablock(s) global, I. DOI: 10.1107/S2056989015004417/wm5132sup1.cif

Structure factors: contains datablock(s) I. DOI: 10.1107/S2056989015004417/wm5132Isup2.hkl

CCDC reference: 1051905

Additional supporting information:  crystallographic information; 3D view; checkCIF report

## Figures and Tables

**Table 1:** Hydrogen-bond geometry (, )

*D*H*A*	*D*H	H*A*	*D* *A*	*D*H*A*
O5H1*O*5O3^i^	0.78(3)	2.00(3)	2.785(3)	175(3)
O5H2*O*5O1^ii^	0.85(4)	2.00(3)	2.845(3)	172(4)
O6H1*O*6O1	0.71(3)	2.15(3)	2.857(3)	170(3)
O6H2*O*6O3^iii^	0.89(3)	1.85(3)	2.736(3)	175(3)
O7H1*O*7O1^iii^	0.64(4)	2.17(3)	2.809(3)	173(4)
O7H2*O*7O4^ii^	0.90(4)	1.83(3)	2.720(3)	168(4)
O8H1*O*8O4^i^	0.76(3)	1.96(3)	2.722(3)	178(4)
O8H2*O*8O2	0.84(3)	1.90(3)	2.737(3)	175(3)
N3H3*N*O2^iv^	0.93(3)	1.93(3)	2.858(3)	178(3)
C4H4O2^iv^	0.93	2.40	3.287(3)	160

Symmetry codes: (i) 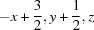; (ii) 
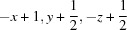
; (iii) 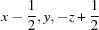; (iv) 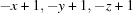.

**Table 2:** Experimental details

Crystal data	
Chemical formula	[Fe(C_8_H_7_N_3_)(H_2_O)_4_]SO_4_
*M*_r_	369.14
Crystal system, space group	Orthorhombic, *P* *b* *c* *a*
Temperature (K)	293
*a*, *b*, *c* ()	12.476(5), 11.741(5), 20.313(7)
*V* (^3^)	2975.5(19)
*Z*	8
Radiation type	Mo *K*
(mm^1^)	1.19
Crystal size (mm)	0.34 0.20 0.11

Data collection	
Diffractometer	Bruker APEXII CCD
Absorption correction	Multi-scan (*SADABS*; Bruker, 2009 ▸)
*T*_min_, *T*_max_	0.802, 0.871
No. of measured, independent and observed [*I* > 2(*I*)] reflections	20168, 4417, 3008
*R*_int_	0.042
(sin /)_max_ (^1^)	0.715

Refinement	
*R*[*F* ^2^ > 2(*F* ^2^)], *wR*(*F* ^2^), *S*	0.036, 0.091, 1.08
No. of reflections	4417
No. of parameters	226
H-atom treatment	H atoms treated by a mixture of independent and constrained refinement
_max_, _min_ (e ^3^)	0.47, 0.41

Computer programs: *APEX2* and *SAINT* (Bruker, 2009 ▸), *SHELXS97* (Sheldrick, 2008 ▸), *SHELXL2014* (Sheldrick, 2015 ▸), *ORTEP-3 for Windows* and *WinGX*(Farrugia, 2012 ▸), *Mercury* (Macrae, 2006 ▸) and *PARST* (Nardelli, 1995 ▸).

## supplementary crystallographic information

### Crystal data

**Table:**

[Fe(C_8_H_7_N_3_)(H_2_O)_4_]SO_4_	*F*(000) = 1520
*M**_r_* = 369.14	*D*_x_ = 1.648 Mg m^−^^3^
Orthorhombic, *P**b**c**a*	Mo *K*α radiation, λ = 0.71073 Å
Hall symbol: -P 2ac 2ab	Cell parameters from 9606 reflections
*a* = 12.476 (5) Å	θ = 2.5–30.0°
*b* = 11.741 (5) Å	µ = 1.19 mm^−^^1^
*c* = 20.313 (7) Å	*T* = 293 K
*V* = 2975.5 (19) Å^3^	Block, yellow
*Z* = 8	0.34 × 0.20 × 0.11 mm

### Data collection

**Table:**

Bruker APEXII CCD diffractometer	4417 independent reflections
Radiation source: fine-focus sealed tube	3008 reflections with *I* > 2σ(*I*)
Graphite monochromator	*R*_int_ = 0.042
φ & ω scans	θ_max_ = 30.5°, θ_min_ = 2.6°
Absorption correction: multi-scan (*SADABS*; Bruker, 2009)	*h* = −17→17
*T*_min_ = 0.802, *T*_max_ = 0.871	*k* = −14→16
20168 measured reflections	*l* = −28→27

### Refinement

**Table:**

Refinement on *F*^2^	226 parameters
Least-squares matrix: full	H atoms treated by a mixture of independent and constrained refinement
*R*[*F*^2^ > 2σ(*F*^2^)] = 0.036	*w* = 1/[σ^2^(*F*_o_^2^) + (0.041*P*)^2^] where *P* = (*F*_o_^2^ + 2*F*_c_^2^)/3
*wR*(*F*^2^) = 0.091	(Δ/σ)_max_ < 0.001
*S* = 1.08	Δρ_max_ = 0.47 e Å^−^^3^
4417 reflections	Δρ_min_ = −0.41 e Å^−^^3^

### Special details

**Table:**

Geometry. All e.s.d.'s (except the e.s.d. in the dihedral angle between two l.s. planes) are estimated using the full covariance matrix. The cell e.s.d.'s are taken into account individually in the estimation of e.s.d.'s in distances, angles and torsion angles; correlations between e.s.d.'s in cell parameters are only used when they are defined by crystal symmetry. An approximate (isotropic) treatment of cell e.s.d.'s is used for estimating e.s.d.'s involving l.s. planes.

### Fractional atomic coordinates and isotropic or equivalent isotropic displacement parameters (Å^2^)

**Table:**

	*x*	*y*	*z*	*U*_iso_*/*U*_eq_	
Fe1	0.45016 (2)	0.72203 (3)	0.35060 (2)	0.02305 (9)	
S1	0.74974 (4)	0.47721 (5)	0.30259 (2)	0.02198 (11)	
O1	0.69197 (10)	0.52744 (13)	0.24568 (6)	0.0295 (3)	
O2	0.69268 (12)	0.50603 (15)	0.36361 (7)	0.0378 (4)	
O3	0.85850 (11)	0.52416 (15)	0.30581 (7)	0.0368 (4)	
O4	0.75368 (13)	0.35340 (15)	0.29605 (7)	0.0441 (4)	
O5	0.45617 (15)	0.89924 (16)	0.33077 (10)	0.0416 (4)	
O6	0.50247 (15)	0.66267 (16)	0.25674 (7)	0.0336 (4)	
O7	0.29469 (14)	0.71843 (19)	0.30864 (8)	0.0322 (4)	
O8	0.61694 (12)	0.72412 (17)	0.37388 (8)	0.0314 (4)	
N1	0.42117 (13)	0.54515 (16)	0.38829 (7)	0.0274 (4)	
N2	0.40687 (14)	0.74690 (16)	0.45132 (8)	0.0294 (4)	
N3	0.35709 (14)	0.6734 (2)	0.54639 (8)	0.0353 (5)	
C1	0.42985 (17)	0.4458 (2)	0.35520 (10)	0.0357 (5)	
H1	0.4548	0.4477	0.3121	0.043*	
C2	0.40365 (18)	0.3420 (2)	0.38200 (11)	0.0405 (6)	
H2	0.4107	0.2753	0.3577	0.049*	
C3	0.36666 (19)	0.3396 (2)	0.44581 (12)	0.0438 (6)	
H3	0.3484	0.2707	0.4654	0.053*	
C4	0.35690 (17)	0.4400 (2)	0.48053 (11)	0.0387 (6)	
H4	0.3314	0.4397	0.5235	0.046*	
C5	0.38537 (15)	0.5406 (2)	0.45065 (9)	0.0279 (5)	
C6	0.38177 (14)	0.6510 (2)	0.48286 (9)	0.0283 (5)	
C7	0.36954 (17)	0.7871 (2)	0.55631 (11)	0.0398 (6)	
H7	0.3593	0.8266	0.5955	0.048*	
C8	0.40013 (17)	0.8319 (2)	0.49715 (10)	0.0362 (5)	
H8	0.4142	0.9085	0.4893	0.043*	
H1O5	0.510 (2)	0.932 (2)	0.3248 (13)	0.049 (8)*	
H2O5	0.408 (3)	0.933 (3)	0.3090 (15)	0.084 (12)*	
H1O6	0.5519 (19)	0.632 (2)	0.2580 (12)	0.036 (8)*	
H2O6	0.453 (2)	0.617 (3)	0.2387 (13)	0.062 (9)*	
H1O7	0.273 (2)	0.672 (3)	0.2986 (13)	0.045 (11)*	
H2O7	0.286 (2)	0.770 (3)	0.2761 (16)	0.066 (10)*	
H1O8	0.652 (2)	0.760 (2)	0.3515 (11)	0.038 (8)*	
H2O8	0.644 (2)	0.658 (3)	0.3708 (13)	0.050 (9)*	
H3N	0.3399 (19)	0.615 (2)	0.5753 (13)	0.054 (8)*	

### Atomic displacement parameters (Å^2^)

**Table:**

	*U*^11^	*U*^22^	*U*^33^	*U*^12^	*U*^13^	*U*^23^
Fe1	0.02282 (15)	0.02469 (18)	0.02163 (14)	−0.00077 (12)	0.00178 (10)	0.00238 (11)
S1	0.0206 (2)	0.0222 (3)	0.0231 (2)	0.00159 (19)	−0.00054 (17)	0.00183 (18)
O1	0.0279 (7)	0.0296 (9)	0.0309 (7)	0.0009 (6)	−0.0064 (5)	0.0067 (6)
O2	0.0427 (9)	0.0408 (11)	0.0300 (7)	0.0072 (8)	0.0144 (6)	0.0084 (7)
O3	0.0214 (7)	0.0493 (12)	0.0397 (8)	−0.0045 (7)	−0.0021 (6)	0.0095 (7)
O4	0.0598 (10)	0.0226 (10)	0.0500 (9)	0.0088 (8)	−0.0224 (8)	−0.0042 (7)
O5	0.0300 (9)	0.0299 (11)	0.0649 (11)	−0.0052 (8)	−0.0073 (8)	0.0177 (8)
O6	0.0263 (8)	0.0449 (12)	0.0296 (8)	0.0023 (9)	0.0008 (6)	−0.0053 (7)
O7	0.0292 (8)	0.0304 (11)	0.0370 (9)	−0.0006 (8)	−0.0061 (6)	−0.0014 (8)
O8	0.0250 (8)	0.0322 (11)	0.0372 (8)	−0.0020 (8)	−0.0001 (6)	0.0057 (8)
N1	0.0290 (9)	0.0306 (11)	0.0226 (8)	−0.0024 (8)	−0.0005 (6)	0.0035 (7)
N2	0.0272 (9)	0.0343 (12)	0.0267 (9)	0.0011 (8)	0.0019 (7)	−0.0027 (7)
N3	0.0344 (10)	0.0474 (14)	0.0241 (9)	−0.0006 (9)	0.0072 (7)	0.0031 (8)
C1	0.0399 (12)	0.0366 (15)	0.0305 (11)	0.0023 (11)	−0.0040 (9)	−0.0014 (9)
C2	0.0422 (13)	0.0323 (15)	0.0470 (13)	−0.0037 (12)	−0.0076 (10)	−0.0068 (11)
C3	0.0430 (14)	0.0397 (17)	0.0486 (14)	−0.0108 (12)	−0.0005 (10)	0.0068 (11)
C4	0.0341 (12)	0.0483 (17)	0.0338 (11)	−0.0112 (11)	0.0032 (9)	0.0090 (10)
C5	0.0209 (9)	0.0370 (14)	0.0257 (9)	−0.0040 (9)	−0.0015 (7)	0.0048 (8)
C6	0.0211 (9)	0.0413 (15)	0.0227 (9)	−0.0017 (9)	0.0033 (7)	0.0050 (8)
C7	0.0352 (12)	0.0516 (18)	0.0326 (11)	0.0047 (11)	0.0045 (9)	−0.0049 (10)
C8	0.0353 (12)	0.0340 (15)	0.0395 (12)	0.0010 (11)	0.0022 (9)	−0.0073 (10)

### Geometric parameters (Å, º)

**Table:**

Fe1—O7	2.1191 (18)	N1—C1	1.351 (3)
Fe1—O5	2.121 (2)	N2—C6	1.333 (3)
Fe1—O6	2.1323 (15)	N2—C8	1.368 (3)
Fe1—O8	2.1340 (17)	N3—C6	1.353 (2)
Fe1—N2	2.1361 (17)	N3—C7	1.359 (3)
Fe1—N1	2.243 (2)	N3—H3N	0.93 (3)
S1—O4	1.4605 (19)	C1—C2	1.373 (4)
S1—O3	1.4661 (16)	C1—H1	0.9300
S1—O2	1.4688 (14)	C2—C3	1.376 (3)
S1—O1	1.4844 (14)	C2—H2	0.9300
O5—H1O5	0.78 (3)	C3—C4	1.379 (4)
O5—H2O5	0.85 (3)	C3—H3	0.9300
O6—H1O6	0.71 (2)	C4—C5	1.374 (3)
O6—H2O6	0.89 (3)	C4—H4	0.9300
O7—H1O7	0.64 (3)	C5—C6	1.453 (3)
O7—H2O7	0.90 (3)	C7—C8	1.366 (3)
O8—H1O8	0.76 (3)	C7—H7	0.9300
O8—H2O8	0.84 (3)	C8—H8	0.9300
N1—C5	1.344 (2)		
			
O7—Fe1—O5	88.60 (8)	C5—N1—C1	117.44 (19)
O7—Fe1—O6	85.07 (7)	C5—N1—Fe1	114.36 (15)
O5—Fe1—O6	98.06 (8)	C1—N1—Fe1	128.10 (14)
O7—Fe1—O8	169.08 (6)	C6—N2—C8	105.96 (18)
O5—Fe1—O8	89.79 (7)	C6—N2—Fe1	113.84 (14)
O6—Fe1—O8	84.46 (7)	C8—N2—Fe1	140.12 (17)
O7—Fe1—N2	99.00 (7)	C6—N3—C7	107.85 (19)
O5—Fe1—N2	93.25 (8)	C6—N3—H3N	120.6 (17)
O6—Fe1—N2	168.09 (7)	C7—N3—H3N	131.4 (17)
O8—Fe1—N2	91.87 (7)	N1—C1—C2	123.3 (2)
O7—Fe1—N1	88.35 (7)	N1—C1—H1	118.3
O5—Fe1—N1	168.26 (7)	C2—C1—H1	118.3
O6—Fe1—N1	92.97 (7)	C1—C2—C3	118.2 (2)
O8—Fe1—N1	95.29 (7)	C1—C2—H2	120.9
N2—Fe1—N1	76.04 (7)	C3—C2—H2	120.9
O4—S1—O3	110.31 (10)	C2—C3—C4	119.5 (2)
O4—S1—O2	108.80 (10)	C2—C3—H3	120.2
O3—S1—O2	108.93 (9)	C4—C3—H3	120.2
O4—S1—O1	109.93 (9)	C5—C4—C3	119.1 (2)
O3—S1—O1	109.55 (9)	C5—C4—H4	120.5
O2—S1—O1	109.29 (9)	C3—C4—H4	120.5
Fe1—O5—H1O5	123 (2)	N1—C5—C4	122.4 (2)
Fe1—O5—H2O5	122 (2)	N1—C5—C6	113.50 (18)
H1O5—O5—H2O5	108 (3)	C4—C5—C6	124.04 (19)
Fe1—O6—H1O6	113.3 (19)	N2—C6—N3	110.3 (2)
Fe1—O6—H2O6	110.5 (17)	N2—C6—C5	122.02 (17)
H1O6—O6—H2O6	108 (3)	N3—C6—C5	127.6 (2)
Fe1—O7—H1O7	122 (3)	N3—C7—C8	106.2 (2)
Fe1—O7—H2O7	113.1 (18)	N3—C7—H7	126.9
H1O7—O7—H2O7	106 (3)	C8—C7—H7	126.9
Fe1—O8—H1O8	115.5 (19)	C7—C8—N2	109.6 (2)
Fe1—O8—H2O8	111.1 (18)	C7—C8—H8	125.2
H1O8—O8—H2O8	104 (3)	N2—C8—H8	125.2

### Hydrogen-bond geometry (Å, º)

**Table:**

*D*—H···*A*	*D*—H	H···*A*	*D*···*A*	*D*—H···*A*
O5—H1*O*5···O3^i^	0.78 (3)	2.00 (3)	2.785 (3)	175 (3)
O5—H2*O*5···O1^ii^	0.85 (4)	2.00 (3)	2.845 (3)	172 (4)
O6—H1*O*6···O1	0.71 (3)	2.15 (3)	2.857 (3)	170 (3)
O6—H2*O*6···O3^iii^	0.89 (3)	1.85 (3)	2.736 (3)	175 (3)
O7—H1*O*7···O1^iii^	0.64 (4)	2.17 (3)	2.809 (3)	173 (4)
O7—H2*O*7···O4^ii^	0.90 (4)	1.83 (3)	2.720 (3)	168 (4)
O8—H1*O*8···O4^i^	0.76 (3)	1.96 (3)	2.722 (3)	178 (4)
O8—H2*O*8···O2	0.84 (3)	1.90 (3)	2.737 (3)	175 (3)
N3—H3*N*···O2^iv^	0.93 (3)	1.93 (3)	2.858 (3)	178 (3)
C4—H4···O2^iv^	0.93	2.40	3.287 (3)	160

Symmetry codes: (i) −*x*+3/2, *y*+1/2, *z*; (ii) −*x*+1, *y*+1/2, −*z*+1/2; (iii) *x*−1/2, *y*, −*z*+1/2; (iv) −*x*+1, −*y*+1, −*z*+1.

## References

[bb1] Addala, A., Setifi, F., Kottrup, K. G., Glidewell, C., Setifi, Z., Smith, G. & Reedijk, J. (2015). *Polyhedron*, **87**, 307–310.

[bb2] Atmani, C., Setifi, F., Benmansour, S., Triki, S., Marchivie, M., Salaün, J.-Y. & Gómez-García, C. J. (2008). *Inorg. Chem. Commun.* **11**, 921–924.

[bb3] Belamri, Y., Setifi, F., Francuski, B. M., Novaković, S. B. & Zouaoui, S. (2014). *Acta Cryst.* E**70**, 544–546. 10.1107/S1600536814024982 PMC4257445 25552988

[bb4] Benmansour, S., Atmani, C., Setifi, F., Triki, S., Marchivie, M. & Gómez-García, C. J. (2010). *Coord. Chem. Rev.* **254**, 1468–1478.

[bb5] Benmansour, S., Setifi, F., Gómez-García, C. J., Triki, S. & Coronado, E. (2008). *Inorg. Chim. Acta*, **361**, 3856–3862.

[bb6] Benmansour, S., Setifi, F., Triki, S. & Gómez-García, C. J. (2012). *Inorg. Chem.* **51**, 2359–2365. 10.1021/ic202361p 22296602

[bb7] Bruker (2009). *APEX2*, *SAINT* and *SADABS*. Bruker AXS Inc., Madison, Wisconsin, USA.

[bb8] Dupouy, G., Marchivie, M., Triki, S., Sala-Pala, J., Gómez-García, C. J., Pillet, S., Lecomte, C. & Létard, J.-F. (2009). *Chem. Commun.* pp. 3404–3406. 10.1039/b902339a 19503885

[bb9] Dupouy, G., Marchivie, M., Triki, S., Sala-Pala, J., Salaün, J.-Y., Gómez-García, C. J. & Guionneau, P. (2008). *Inorg. Chem.* **47**, 8921–8931. 10.1021/ic800955r 18686945

[bb10] Farrugia, L. J. (2012). *J. Appl. Cryst.* **45**, 849–854.

[bb11] Macrae, C. F., Edgington, P. R., McCabe, P., Pidcock, E., Shields, G. P., Taylor, R., Towler, M. & van de Streek, J. (2006). *J. Appl. Cryst.* **39**, 453–457.

[bb12] Miyazaki, A., Okabe, K., Enoki, T., Setifi, F., Golhen, S., Ouahab, L., Toita, T. & Yamada, J. (2003). *Synth. Met.* **137**, 1195–1196.

[bb13] Nardelli, M. (1995). *J. Appl. Cryst.* **28**, 659.

[bb14] Setifi, Z., Domasevitch, K. V., Setifi, F., Mach, P., Ng, S. W., Petříček, V. & Dušek, M. (2013). *Acta Cryst.* C**69**, 1351–1356. 10.1107/S0108270113027108 24192188

[bb15] Setifi, F., Golhen, S., Ouahab, L., Turner, S. S. & Day, P. (2002). *CrystEngComm*, **4**, 1–6.

[bb16] Setifi, Z., Lehchili, F., Setifi, F., Beghidja, A., Ng, S. W. & Glidewell, C. (2014). *Acta Cryst.* C**70**, 338–341. 10.1107/S2053229614004379 24594730

[bb17] Sheldrick, G. M. (2008). *Acta Cryst.* A**64**, 112–122. 10.1107/S0108767307043930 18156677

[bb18] Sheldrick, G. M. (2015). *Acta Cryst.* C**71**, 3–8.

[bb19] Tinant, B., Decamp, C., Robert, F. & Garcia, Y. Z. (2010). *Z. Kristallogr. New Cryst. Struct.* **225**, 729–732.

